# Spatiotemporal changes, trade-offs, and synergistic relationships in ecosystem services provided by the Aral Sea Basin

**DOI:** 10.7717/peerj.12623

**Published:** 2021-12-16

**Authors:** Chao liang Chen, Xi Chen, Jing Qian, Zengyun Hu, Jun Liu, Xiuwei Xing, Duman Yimamaidi, Zhanar Zhakan, Jiayu Sun, Shujie Wei

**Affiliations:** 1 State Key Laboratory of Desert and Oasis Ecology, Xinjiang Institute of Ecology and Geography, Chinese Academy of Sciences, Urumqi, China; 2 Research Center for Ecology and Environment of Central Asia, CAS, Urumqi, China; 3 Shenzhen Institutes of Advanced Technology, Chinese Academy of Sciences, Shenzhen, China; 4 University of Chinese Academy of Sciences, Beijing, China; 5 Key Laboratory of GIS & RS Application Xinjiang Uygur Autonomous Region, Urumqi, China; 6 TripleSAI Technology, Shenzhen, China; 7 Kazahk Agro Technical University named Saken Seifullin, Nur-sultan, Kazakstan

**Keywords:** Ecosystem services, LULC change, Trade-off and synergy, Aral Sea basin, PLUS model

## Abstract

Intense human activities in the Aral Sea Basin have changed its natural distribution of land use. Although they provide certain economic benefits, these anthropogenic influences have led to the rapid shrinkage of the Aral Sea, severely affecting the region’s ecosystem. However, the spatiotemporal variability of the Aral Sea Basin’s Ecosystem Service Values (ESVs) is not well understood. In this study, we used 300-meter resolution land use maps from 1995, 2005, and 2015 and the Patch-generating Land Use Simulation (PLUS) model to predict the future land use patterns of the Aral Sea Basin in 2025. Simultaneously, we divided the Aral Sea Basin into three regions (upstream, midstream, and downstream) and evaluated the dynamic responses of their ESVs to Land Use and Land Cover (LULC) changes. The changes in the types of ecosystem services provided by the Aral Sea Basin, their trade-off, and synergistic relationships were analyzed by weighting their associations. The results showed that from 1995 to 2025, the grassland, urban, and cropland areas in the Aral Sea Basin will expand rapidly, while the areas covered by water bodies will shrink rapidly, causing a total loss of 31.97 billion USD. The downstream loss of 27.79 billion USD of the total amount is mainly caused by the conversion of water bodies to bare land. The ESVs of the middle region will increase by 6.81 billion USD, mainly due to the large amount of water extracted from the Amu Darya and Syr Darya Rivers in the middle regions of the Aral Sea Basin that are used to reclaim cultivated land and expand urban areas. The ESVs and areas experiencing land use changes in the upper regions are relatively small. At the same time, our results show that biodiversity, food production, and water regulation are the major ecosystem service functions, and account for 79.46% of the total ESVs. Of the ecosystem service relationships in the Aral Sea Basin, synergy accounts for 55.56% of the interactions, with a fewer amount of trade-off exchanges. This synergy mainly exists in the relationships involving water regulation, waste treatment and recreation, and culture and tourism. We propose protection measures that will coordinate eco-environmental protection efforts with socioeconomic development in the region in order to achieve the United Nations’ sustainable development goals.

## Introduction

Ecosystems provide many services to humans that contribute to both well-being and economic wealth (Allison & Organization, 2006; Costanza et al., 1997). Ecosystem services refer to the natural utilities that humans rely on for survival that are formed and maintained by ecosystems and ecological processes. They include provisional, regulatory, supporting, and cultural services (Costanza et al., 1997; Costanza et al., 2014). Different types of services contribute differently to society. In order to coordinate the development of various services, it is necessary to adopt a unified evaluation standard to measure the value of each ecosystem service (Metzger et al., 2006). Measuring economic service values (ESVs) in monetary units provides an integrated and universal method for evaluating the value of ecosystem services and directly reflects the direction and speed of changes in ESVs (Bryan, Ye & Connor, 2018; De Groot et al. 2012; Gomez-Baggethun & Barton, 2013). Additionally, these values can help policymakers quickly integrate regional resources and make the best decisions to reasonably allocate regional resources (Farley, 2008).

A variety of methods and models such as cost-based methods, the Benefit Transfer Method (BTM), energy analysis models, the Integrated Valuation of Ecosystem Services and Trade-offs (InVEST) model, and the Social Value of Ecosystem Services (SolVES) model have been studied by researchers to quantify the dynamic response of land use to ESVs (Bagstad, Semmens & Winthrop, 2013; Redhead et al., 2016; Sherrouse, Semmens & Clement, 2014; Shi et al., 2020; Zhan et al., 2019). The most effective approach is the BTM, which was developed by Costanza et al. (1997) to estimate global ESVs. This approach uses a value matrix derived from either a single case study or multiple studies to calculate the value of each ecosystem service function (Costanza et al., 1997; Shi et al., 2020). Changes in physical supply have been typically assessed using land-use dynamics that act as a proxy for the spatial distribution of ecosystem service-producing units, or by directly modeling the production of ecosystem services themselves (Bateman et al., 2013; Bryan, Ye & Connor, 2018; Costanza et al., 2014). Therefore, changes in land use types directly lead to fluctuations in the value of services provided to humans by ecosystems. Numerous studies have shown that unsuitable land use strategies cause serious ecological degradation; for example, excessive urban expansion and the conversion of deforested land to cultivated land leads to land degradation and the reduced supply of raw materials (Metzger et al., 2006; Polasky et al., 2011).

The Aral Sea Basin is located in an arid area in the middle of Central Asia, where the Syr Darya, Amu Darya, and their surrounding reservoirs and lakes constitute the main surface water resources. This flow is substantially diminished by evaporation, transpiration from vegetation along the banks, and bed filtration as the rivers pass across the desert to the Aral Sea. Additionally, the development of modern large-scale irrigation has caused the average inflow of the Amu Darya into the Aral Sea to decrease from 18.2 km^3^ in 1990 to about 5.3 km^3^ in 2015, and the Syr Darya has decreased from 5.8 km^3^ in 1990 to about 4.4 km^3^ in 2015. Therefore, the redistribution of surface water resources is the main cause of land use and land cover (LULC) changes in the Aral Sea Basin (Bai et al., 2012; Breckle et al., 2001; Kulmatov et al., 2018). The Aral Sea was the world’s fourth largest lake prior to 1960, but it has shrunk significantly as surface water sources have been diverted for irrigation purposes over the past five decades, notably following the collapse of the Soviet Union in the early 1990s (Deliry et al., 2020; Hamidov, Helming & Balla, 2016). Falling lake levels and surface area reductions have had profound effects on this fragile ecological environment. Moreover, the region’s climatic conditions have also changed drastically, and temperatures in the Aral Sea Basin have been rising at a speed of 3.2 °C/decade since 1960 (Massakbayeva et al., 2019). At the same time, precipitation levels have exhibited a significant upward trend at a rate of 0.8 mm/decade (Li et al., 2015; Massakbayeva et al., 2019). Therefore, knowledge of the current changes in and dynamics of ESVs in relation to climatic changes and anthropogenic activities is important for ecological monitoring and sustainability management in the Aral Sea Basin. Previous studies have evaluated the ecosystem service functions and values for all of Central Asia. For example, Chen et al. (2013) evaluated the spatial and temporal changes in LULC, net primary productivity (NPP), actual evapotranspiration (AET), and crop production in Central Asia between 1990 and 2009, and Li et al. (2019) assessed the ESVs of Central Asia from 1995 to 2035 using the BTM. However, no studies have specifically evaluated ESVs in the Aral Sea Basin, nor conducted a systematic analysis of the value of ecosystem services in the middle and lower reaches of the Aral Sea.

The aims of this study were to: (1) estimate and project the LULC changes occurring in the Aral Sea Basin from 1995 to 2025, (2) evaluate changes in ESVs in response to LULC changes and reveal the spatial distribution characteristics of those changes in the Aral Sea Basin, and (3) analyze the trade-offs and synergy between various types of ecosystem services across different periods. Knowledge of the variation of ESV characteristics will help promote the rational use of water resources in this region and maintenance of ecological environment protection efforts impacted by climate change and human activities.

## Materials & Methods

### Study area

**Figure 1:**
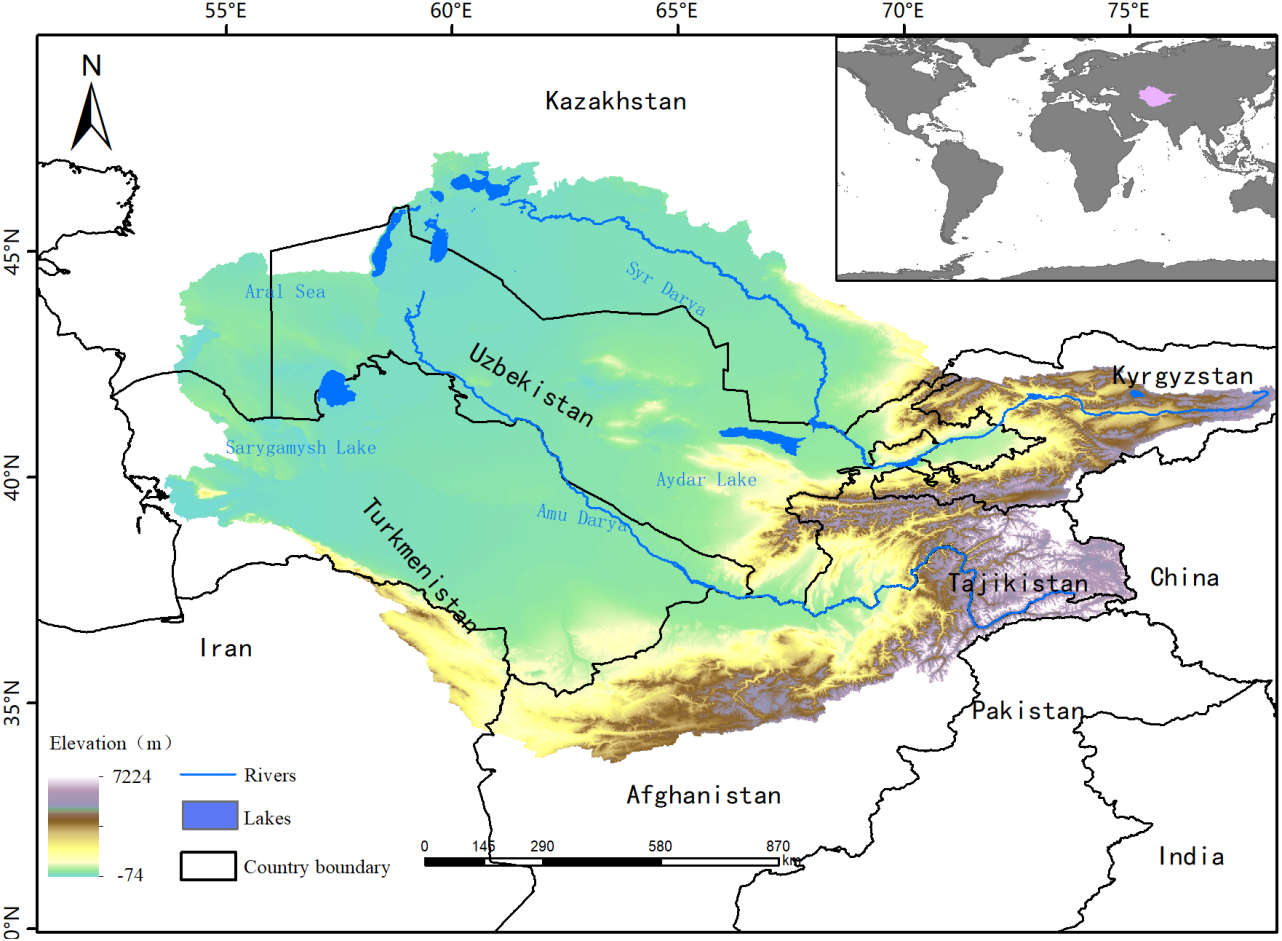
Map showing the location of the Aral Sea Basin.

The Aral Sea Basin (53°37′–78°21′, 33°48′–47°12′) covers 1.7 million square kilometers of Central Asia and is shared by Afghanistan, Iran, Turkmenistan, Kazakhstan, Tajikistan, Uzbekistan, and Kyrgyzstan (Deliry et al., 2020; Harriman, 2014; Fig. 1). The surface water resources in our study area entirely depended on the run-off from the Syr Darya and Amu Darya Rivers, which begin in the Pamir and Tian Shan Mountains (Lioubimtseva, 2015). Amu Darya, the most important river within the Aral Sea Basin, flows nearly 2,400 km from the mountains with an average annual flow of around 79 km^3^ from the drainage basin (Nezlin, Kostianoy & Lebedev, 2004). The total length of the Syr Darya River is 2,500 km and the average annual flow is 37 km^3^ (Nezlin, Kostianoy & Lebedev, 2004). The climate of the Aral Sea Basin is semiarid to arid with cold winters and hot summers (Nezlin, Kostianoy & Li, 2005). The annual precipitation in the region reaches 140 mm/year, and most of the region experiences a distinctive spring maximum (Deliry et al., 2020). Due to the obvious continental climate characteristics in this area, there is a large temperature difference between winter and summer. In 1960, the Aral Sea covered an area of 68,000 km^2^, but it has since shrunk significantly because of the construction of canals from the Amu Darya and Syr Darya basins used to irrigate cultivated land. The potential evapotranspiration has increased in this region over recent decades, exacerbating the water shortage problem (Jiang et al., 2019).

### Data source

Developments in earth observation technology have led to the use of a variety of land use datasets to study land use changes, but these current generated global land cover datasets have almost no consistency in their observation time periods, spatial resolution, or accuracy standards (Poulter et al., 2015). For example, the Copernicus Global Land Cover layers have a higher resolution (100 m), but only cover 2015–2019, and the Moderate Resolution Imaging Spectroradiometer (MODIS) Land Cover Type Product (MCD12Q1) provides global land cover types at yearly intervals (2001–2016), but its resolution is only 500 m. In order to resolve these shortcomings, the European Space Agency (ESA) consistently maps moderate-resolution global land cover over time using surface reflectance from the Medium-Spectral Resolution Imaging Spectrometer (MERIS) aboard the Environmental Satellite (ENVISAT) and Systeme Probatoire d’Observation de la Terre (SPOT) 4 and 5. To validate the data, Bontemps et al. (2013) used the GlobCover 2009 verification database and found that the overall accuracy of the ESA’s Climate Change Initiative Land Cover (CCI-LC) maps was 75.4%. Reinhart et al. (2021) evaluated the consistency between datasets through proportional area comparison, and found that the overall accuracy of the ESA CCI-LC with Coordination and Information on the Environment (CORINE) Land Cover (CLC) reached ∼76%. Although the dataset divided the world into 37 land use types, to match Costanza’s biomes and consider the actual conditions present within the study area, we classified 29 land use types in the study area into seven major LULC types: cropland, forestland, grassland, wetland, urban, bare land, and water (including snow and ice) (Table S1).

Digital elevation data, obtained from the National Aeronautics and Space Administration (NASA) Shuttle Radar Topographic Mission (SRTM) at a spatial resolution of 90 m, were utilized to extract the stream network and contour lines of the Aral Sea Basin (Table 1). They were acquired with the same sensor in a single mission and produced with a single technique: Synthetic Aperture Radar (SAR) interferometry. The absolute and relative vertical accuracies of the Digital Elevation Model (DEM) were ±16 m and ±6 m, respectively (Rabus et al., 2003).

**Table 1:** Data descriptions and resources.

Layer name	Data source	Resolution	Period	Formats
LULC	http://maps.elie.ucl.ac.be/CCI/viewer	300 m	1995–2020	Raster
DEM	http://www2.jpl.nasa.gov/srtm/	90 m	——	Raster
Annual mean precipitation	https://www.worldclim.org/	1000 m	1970–2000	Raster
Annual temperature	https://www.worldclim.org/	1000 m	1970–2000	Raster
Population	https://www.worldpop.org/	1000 m	2015	Raster
Roads	http://sedac.ciesin.columbia.edu/about	——	2014	Vector

In addition, we also collected nine variables, including topographical, population, and land cover parameters, to simulate the contribution of various factors to land expansion (Table 1). The precipitation and temperature data for current climate conditions were extracted from the WorldClim global normal climate (the average for 1970–2000) database with a 30-arc-second (∼1 km) resolution (Fick & Hijmans, 2017). Population data were obtained from the WorldPop program with a resolution of 30-arc-seconds (∼1 km). The road information was extracted from the Socioeconomic Data and Applications Center (SEDAC) managed by the NASA Earth Science Data and Information System (ESDIS) project (Sherbinin et al., 2002). The water surface and slope data were extracted from the ESA CCI-LC maps and DEM, respectively. ArcGIS’s spatial analysis function was used to calculate the Euclidean distance between the water body and the roads, and all variables were resampled to a 1 km resolution. The data for all environmental variables were then resampled to a 1 km × 1 km resolution to match the land use simulation model.

### Model projection of future LULC changes

In order to improve the geographic cellular automata model in the conversion rule mining strategy and landscape dynamic change simulation strategies, Liang et al. (2021) developed the patch-generating land use simulation (PLUS) model, which was based on a comprehensive analysis of the influence of man-made and natural factors.

**Figure 2:**
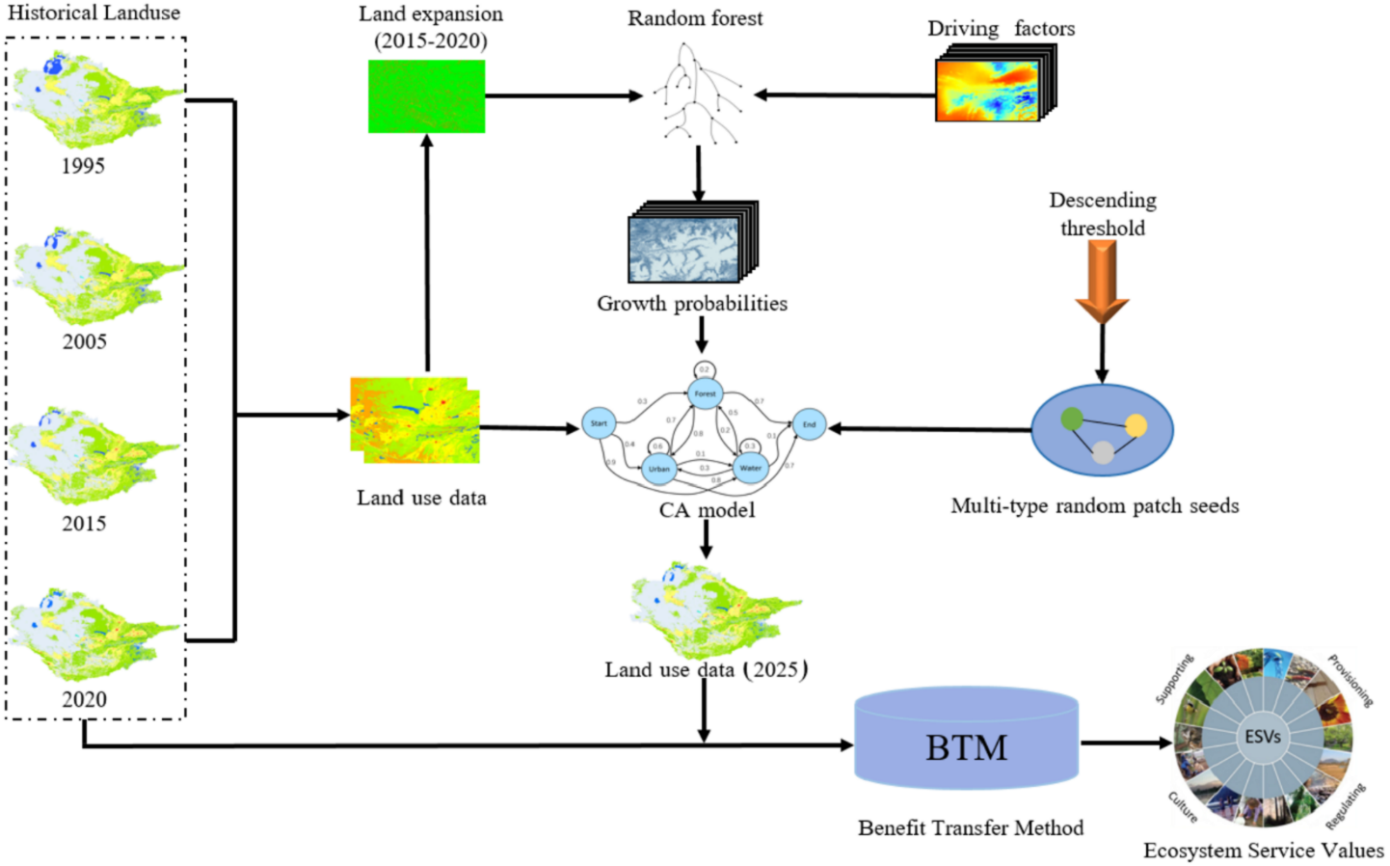
The framework of the PLUS-BTM-ESVs model.

The model proposes a rule mining framework based on the Land Expansion Analysis Strategy (LEAS), which comprehensively considers factors that affect land use changes, such as population, economy, traffic topography, and climate background, so that it can better simulate the LULC pattern with scattered patches (Fig. 2; Li et al., 2021).

**Figure 3:**
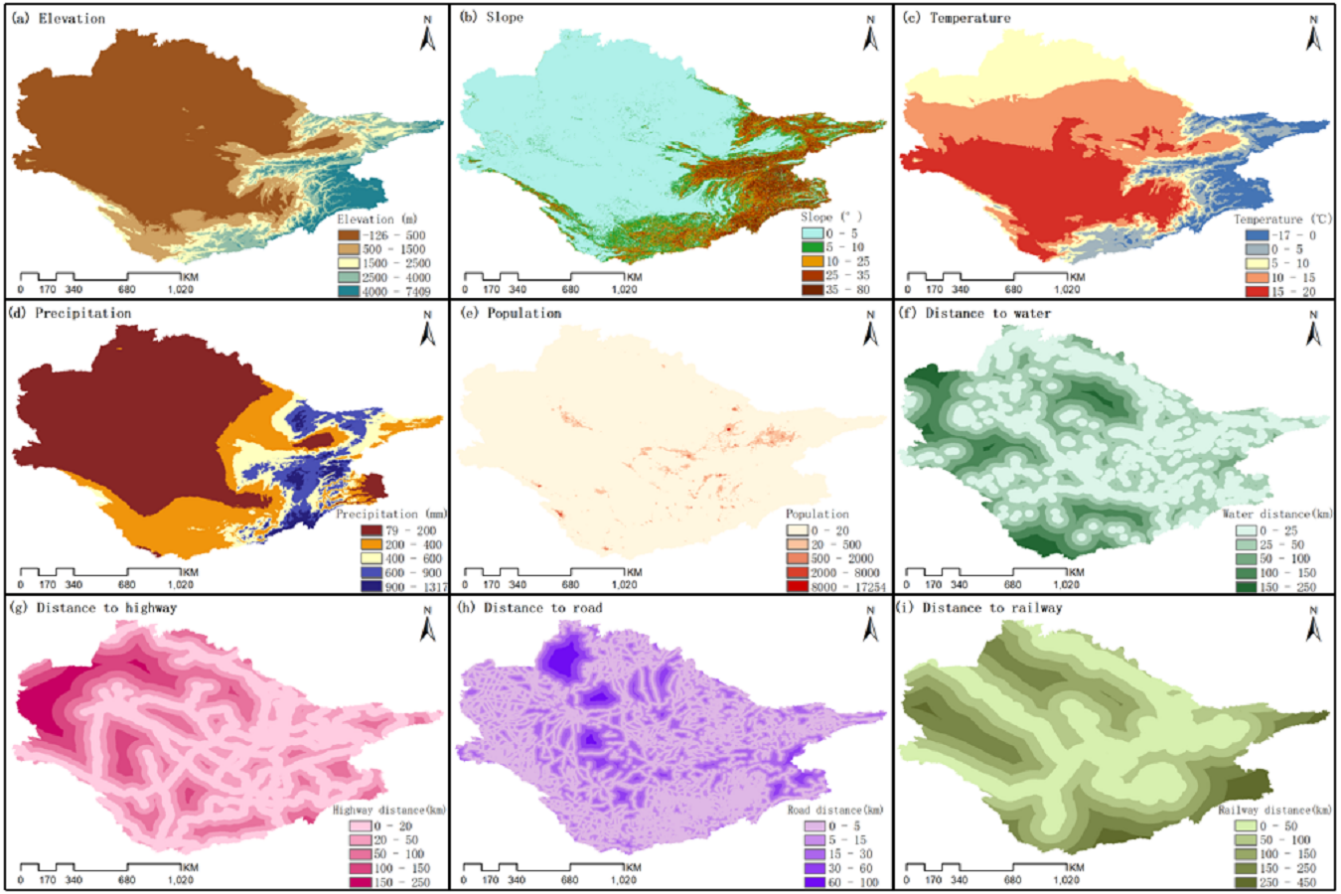
Driving factors affecting land use distribution.

The land use data from the two periods, 2015 and 2020, were overlaid together and the cells were extracted with the changed states from the later date, which represented the change regions for each land use type. Sampling points were randomly selected and divided into subsets according to their land use types, which were then analyzed separately using Random Forest Classification (RFC) algorithms to explore the relationship between the growth of each land use type and multiple driving factors (elevation, slope, temperature, precipitation, population, distance to water, distance to highway, distance to road, and distance to railway) (Fig. 3). On this basis, we proposed a cellular automata (CA) model based on multi-type Random Patch Seeds (CARS) to better simulate the fine-scale patch growth of various LULC types.

A multi-type random patch seeding mechanism based on threshold descent was used in the PLUS model in order to predict the patch evolution for various land-use type scenarios (Shi et al., 2021). By applying a Monte Carlo approach, the probability surface ${P}_{i,k}^{d=1}$ for each land-use type was determined when the neighborhood effect of land use *k* was 0,

\begin{eqnarray*}O{P}_{i,k}^{d=1,t}= \left\{ \begin{array}{@{}ll@{}} \displaystyle {P}_{i,k}^{=1}\times \left( r\times {\mu }_{k} \right) \times {D}_{k}^{t} &\displaystyle  \mathrm{if} {\Omega }_{i,k}^{t}=0 \mathrm{and} r\lt {P}_{i,k}^{d=1}\\ \displaystyle {P}_{i,k}^{d=1}\times {\Omega }_{i,k}^{t}\times {D}_{k}^{t} &\displaystyle  \text{all others}  \end{array} \right. \end{eqnarray*}

where *r* is a random value ranging from 0 to 1; *μ*_*k*_ is the threshold used to generate the new land use patches for land use type *k*, which was determined by the model users. Seeds can create new forms of land use and expand into a series of new patches. If the new land-use type won in a round, a declining threshold *τ* was used to determine the candidate land-use type c chosen by the roulette wheel as follows:

\begin{eqnarray*}\begin{array}{@{}cc@{}} \displaystyle &\displaystyle  \mathrm{If} \sum _{k=1}^{N} \left\vert {G}_{c}^{t-1} \right\vert -\sum _{k=1}^{N} \left\vert {G}_{c}^{t} \right\vert \lt  Step~\text{Then},l=l+1\\ \displaystyle &\displaystyle \left\{ \begin{array}{@{}ll@{}} \displaystyle  \text{Change}  &\displaystyle {P}_{i,c}^{d=1}\gt \tau  \mathrm{and} T{M}_{k,c}=1\\ \displaystyle  \mathrm{No} \text{change}  &\displaystyle {P}_{i,c}^{d=1}\leq \tau  \mathrm{or} T{M}_{k,c}=0 \end{array} \right. \tau ={\delta }^{l}\times r1 \end{array} \end{eqnarray*}

where Step is the step size of the PLUS model used to approximate the land use demand; *δ* is the decay factor of the decreasing threshold *τ*, which ranges from 0 to 1 and is set according to the expert; *r*1 is the normally distributed stochastic value with a mean of 1, ranging from 0 to 2; and *l* is the number of decay steps. *TM*_*k*,*c*_ is the transition matrix that defines whether land use type *k* is allowed to convert to type *c* (Verburg & Overmars, 2009). This study’s neighborhood size was 3. We set the declining threshold *τ* and decay factor *δ* as 0.7 and 0.2, respectively.

To ensure the reliability of the simulation results, we used the Kappa coefficient and Figure of Merit (FoM) to measure the goodness of fit for land use simulation. The calculation formula can be described as follows (Mitsova, Shuster & Wang, 2011):

\begin{eqnarray*}\text{kappa}= \frac{{p}_{0}-{p}_{c}}{1-{p}_{c}} \end{eqnarray*}

where kappa is the simulation accuracy index, *p*_*c*_ is the expected simulation accuracy in a random state, and *p*_0_ is the actual simulation accuracy.

\begin{eqnarray*}\mathrm{FoM}=\mathrm{B}/(\mathrm{A}+\mathrm{B}+\mathrm{C}+\mathrm{D}) \end{eqnarray*}

where A refers to the error area in which the observed change is simulated as persistence, B refers to the correct area in which the observed change is simulated as change, C refers to the error area in which the observed change is simulated as change in the wrong classification, and D refers to the error area in which the observed persistence is simulated as change.

Previous research has demonstrated that the PLUS model can achieve good results in predicting land use changes (Liang et al., 2021) and that its simulation accuracy is better than that of the Future Land Use Simulation (FLUS) model, the Conversion of Land Use and its Effects (CLUE) model, and the Artificial Neural Networks–Cellular Automata (ANN-CA) (Li et al., 2021). In this study, we used the transition probability matrix from 2005 to 2010 to develop the 2015 LULC map (Table S2). The forecast data in 2015 was compared with the actual respective data to verify the model’s accuracy. The simulation performance evaluation showed that the kappa coefficient was 0.92 and the FoM was 0.24, which suggested that the simulation results were accurate and reliable. Then, we used the 2015 and 2020 maps and the FLUS model in the transition probability matrix to predict the 2025 LULC maps (Table S3).

### Assessment of ESVs

In this study, nine ESVs, proposed by Xie et al. (2008), were calculated using the BTM. As the Aral Sea Basin is located in Central Asia, and 83.2% of the area is shared by Turkmenistan, Kazakhstan, Tajikistan, Uzbekistan and Kyrgyzstan, so the equivalent value coefficients of each ES referred to those proposed by Li et al. (2019; Table 2), which were derived from the value coefficient matrix proposed by Costanza et al. (Costanza et al., 2014). We used the assessment model proposed by Costanza et al. (Costanza et al., 1997) as a basis to evaluate the ESVs of the Aral Sea Basin.

\begin{eqnarray*}ES{V}_{t}=\sum {A}_{k}\times {V}_{k} \end{eqnarray*}

\begin{eqnarray*}{V}_{k}=\sum _{i=1}^{n}ES{V}_{ki} \end{eqnarray*}

**Table 2:** The value coefficient of ecosystem services across seven LULC categories in Aral Sea Basin. (US$/ha/yr) reference from Li et al. (2019).

Service type	Sub-type	Cropland	Forestland	Grassland	Wetland	Urban	Bare land	Waterbodies
Provisioning	Food production	2,323	299	1,192	614	0	0	106
	Raw material	219	181	54	539	0	0	0
Regulating	Gas regulation	0	0	9	0	0	0	0
	Climate regulation	411	152	40	3,474	905	0	0
	Water regulation	400	191	63	6,014	16	0	9,322
Supporting	Soil-formation and retention	639	107	46	4,320	0	0	0
	Waste-treatment	397	120	75	3,015	0	0	918
	Biodiversity	1,096	1,097	2,494	3,502	0	0	0
Culture	Recreation, cultural and tourism	82	990	193	4,203	5,740	0	2,166
Total		5,567	3,137	4,166	25,681	6,661	0	12,512

where *ESV*_*t*_ is the total ESVs, *A*_*k*_ is the area of the LULC type *k*, *V*_*k*_ is the ESV of LULC type *k*, and *ESV*_*ki*_ is the *i* type of ESV of LULC type *k*.

The changes in ESVs in the study area were calculated using the following equation derived from a study by Song & Deng (2017).

\begin{eqnarray*}C{R}_{i}= \frac{ES{V}_{end}-ES{V}_{start}}{ES{V}_{start}} \times 100\text{%} \end{eqnarray*}

where *CR*_*i*_ is the change in the ESVs in grid *i*, *ESV*_*end*_ is the ESVs at the end of the study period for grid *i*, and *ESV*_*start*_ is the ESVs at the beginning of the study period.

### Elasticity of ESV changes in relation to LULC

In economics, elasticity is a measure of how responsive one economic variable is to changes in another (Song & Deng, 2017). In this study, we aimed to investigate the responses of ESVs to LULC. We used a sensitivity analysis to evaluate changes in the ESVs in response to 50% ESV coefficient adjustments for each LULC type (Kindu et al., 2016). The formula for calculating the sensitivity coefficient refers to the standard economic concept of elasticity:

\begin{eqnarray*}\mathrm{CS}= \frac{(ES{V}_{j}-ES{V}_{i})/ES{V}_{i}}{(ES{V}_{jk}-ES{V}_{ik})/ES{V}_{ik}} \end{eqnarray*}

where CS is the coefficient of sensitivity; *i* and *j* represent the initial and adjusted values, respectively; and *k* is the LULC category. If *CS* was greater than one, then the estimated ecosystem value was considered elastic relative to that coefficient. However, if *CS* was less than one, then the estimated ecosystem value was considered inelastic and the results were reliable even if the value coefficient had a relatively low accuracy (Kindu et al., 2016; Kreuter et al., 2001).

### Correlation analysis

Correlation analysis can be used to quantitatively describe the degree of linear correlation and clarify the direction of correlation between two variables (Liu, Wu & Chen, 2018). The larger the value, the stronger the correlation and the smaller the value, the weaker the correlation. When the result is greater than 0, this suggests that as one variable increases, the other also increases, making it a coordinated relationship. When the result is less than 0, there is a trade-off relationship. The formula is as follows:

\begin{eqnarray*}{R}_{xy}= \frac{\sum _{i=1}^{n}({x}_{i}-\bar {x})({y}_{i}-\bar {y})}{\sqrt{\sum _{i=1}^{n}({x}_{i}-\bar {x})^{2}}\sqrt{\sum _{i=1}^{n}({y}_{i}-\bar {y})^{2}}} \end{eqnarray*}

where *R*_*xy*_ is the correlation coefficient, n is the number of samples, *x*_*i*_*y*_*i*_ are the ith value of x and y respectively, and $\bar {x}$ and $\bar {y}$ are the average values of x and y, respectively.

## Results

### LULC change detection analysis

**Figure 4:**
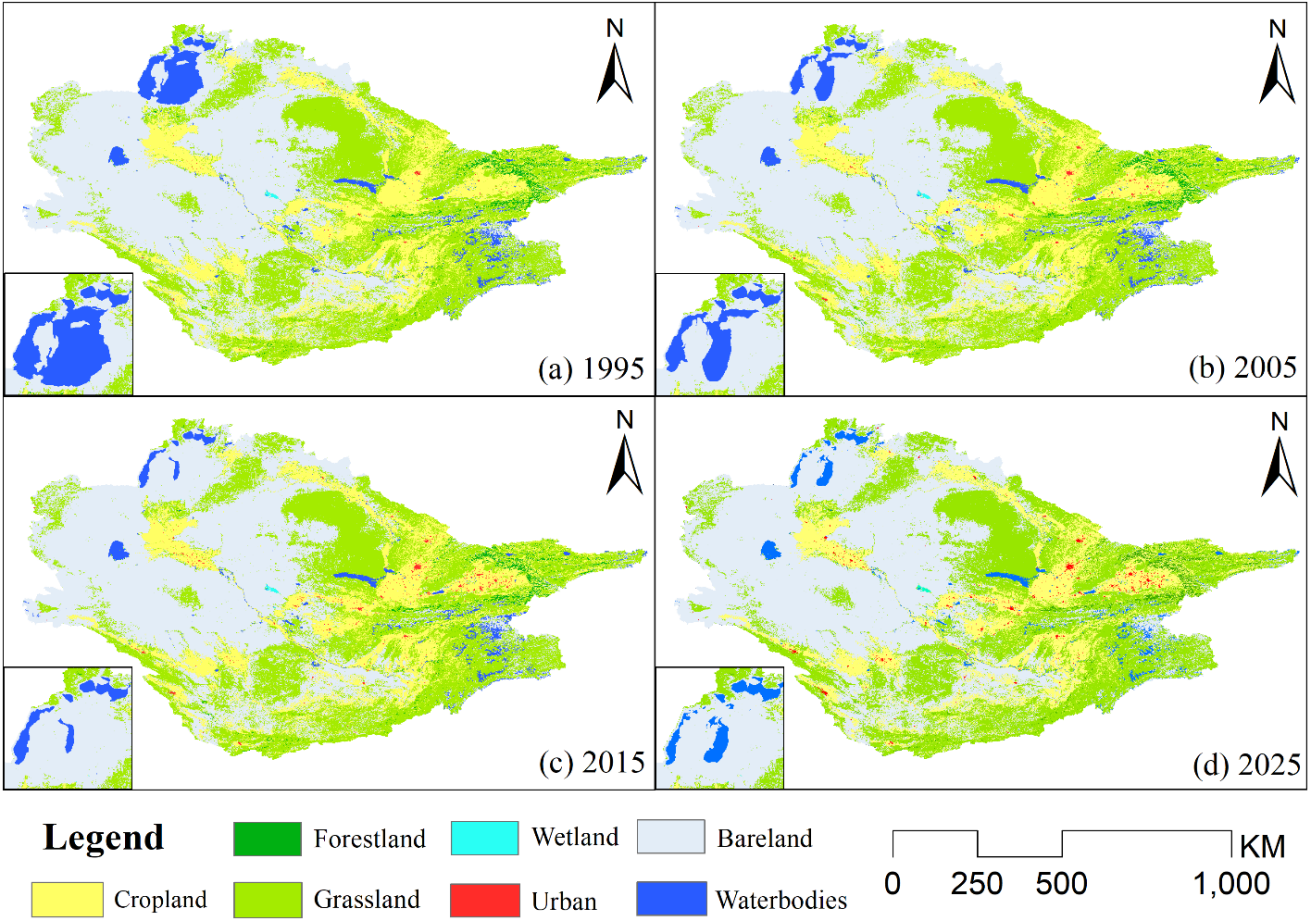
Spatial distribution of LULC changes in the Aral Sea Basin.

Figure 4 shows the spatial distribution of land use in the Aral Sea basin from 1995 to 2025. From 1995 to 2025, the largest cover type in the study area is bare land, accounting for approximately 45.3% of the total study area, followed by grassland and cropland, which account for 33.5% and 17.3%, respectively (Table 3). Among all types of land use, bare land increased the most. The total area increased by 111.44 × 10^4^ ha, and urban land increased by 546.54%. The average annual urban land notably increased by 18.2% from 1995 to 2025. The area of cultivated land increased by 15.2 × 10^4^ ha, but it tended to be stable from 2005 to 2015. It is worth noting that water bodies experienced the largest reduction in area, decreasing by 42.42% from 1995 to 2025, with an average annual reduction of 1.62 × 10^4^ ha. The forestland area decreased by 9.45 × 10^4^ ha. The wetland area has not significantly fluctuated in recent years.

**Figure 5:**
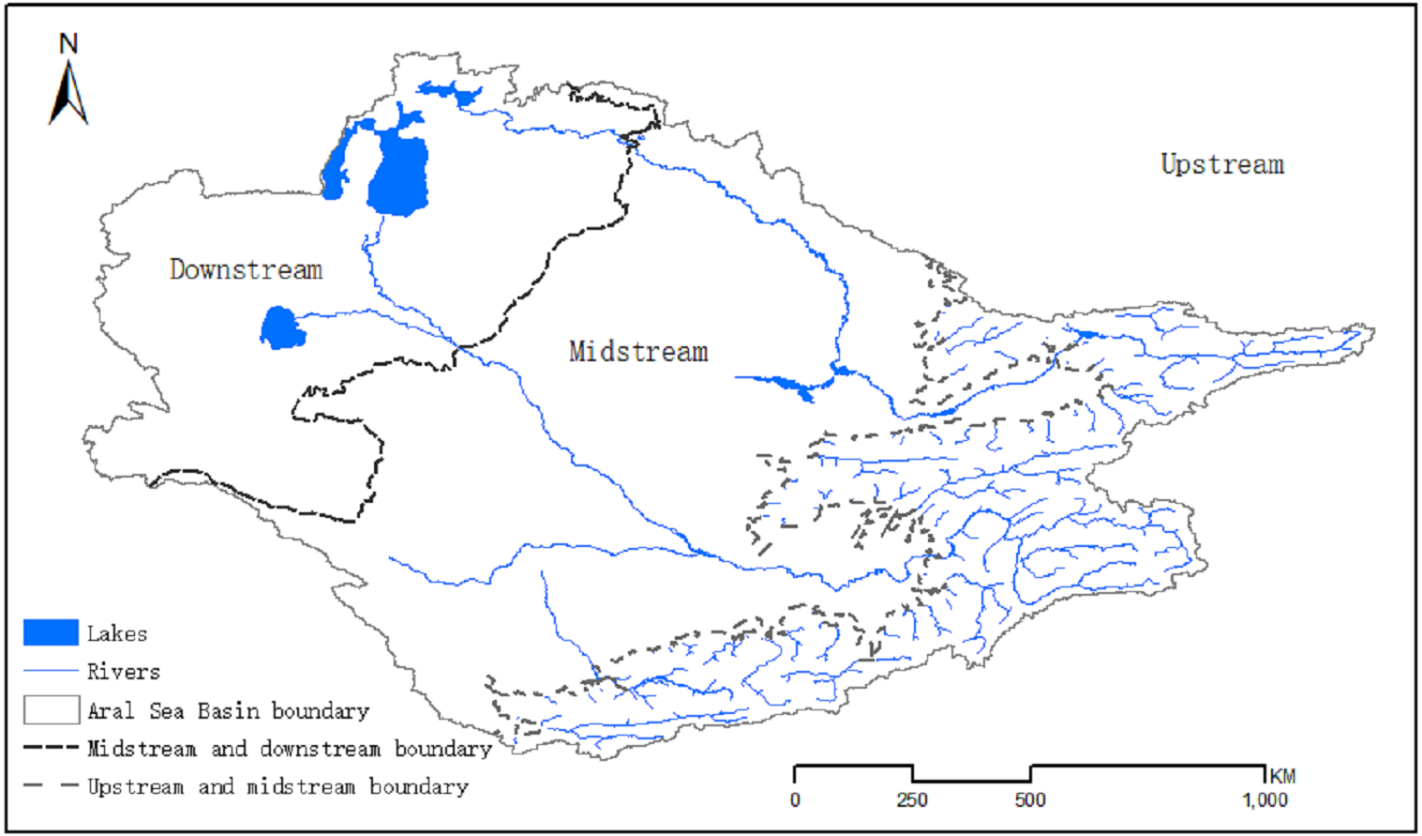
Geographical distribution of the upper, middle and lower reaches of the Aral Sea Basin.

Using the geospatial analysis software ArcGIS, combined with a digital elevation model map, we extracted the natural river network structure in the basin and contour lines within the basin with a distance of 100 m. Through our analysis, we found that the 1,000-meter contour line acted as the dividing line between the river and the alluvial plain of the mountain pass, and the dissipation area of the river was completely below the 100-meter contour line. Therefore, the 1,000-meter and 100-meter contour lines were used as the dividing lines of the upper, middle, and downstream parts of the river, and the land use changes in the upper, middle, and lower reaches were counted separately (Fig. 5).

The area of urban land in the upstream, midstream, and downstream watersheds is predicted to increase rapidly by 622.39%, 536.99%, and 608.98%, respectively (Table S4). Correspondingly, the area of cropland and grassland is also increasing. However, the forestland, wetland, bare land, and water bodies show obvious spatial differentiation. The forestland and water body areas in the upstream and downstream have shown an overall decreasing trend, while the middle regions are increasing. The wetland and bare land areas are constantly decreasing in the upstream area, but are increasing in the downstream reaches. According to the conversion relationship between the different land use types, the upstream forestland and water bodies have been transformed into grassland and cultivated land. In the middle reaches, excluding the shrinking bare land and wetlands, the other land use types have increased to varying degrees. The areas covered by water bodies in the downstream areas have been greatly reduced, and most have been converted into bare land. Grassland, urban land, cropland, and wetland areas have also expanded to varying degrees, while forestland has slightly decreased in area.

### Changes in total ESVs

**Figure 6:**
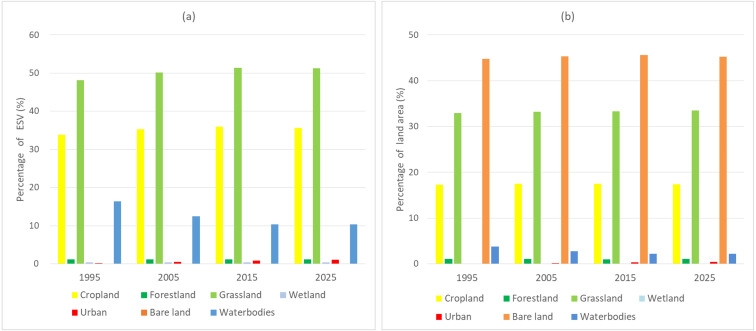
(A) The percentage of ESVs of different land use types; (B) the percentage of land use area.

According to our estimation, the total ESVs of the Aral Sea basin in 1995 was approximately 492.55 billion USD (Table 4). Grasslands constituted the highest contribution at 48.08%, followed by cropland and water body areas (33.79% and 16.39%, respectively) (Fig. 6). Due to the observed LULC changes, the regional ESVs decreased by 16.55 billion USD from1995 to 2005, which can be mainly attributed to the decreased ESVs associated with water bodies that counteracted the ESV gains from cropland, grassland, and urban areas. The regional ESVs further decreased by 9.36 billion USD from 2005 to 2015. Similarly, the decrease in the water ESVs was the main reason for the decrease in the regional ESVs. However, the regional ESVs are predicted to increase by 3.03 billion USD from 2015 to 2025. This is mainly because the ESVs of the water bodies will no longer decline, and the ESVs of the grassland and urban areas show an upward trend, which will overcompensate for the ESV losses associated with cropland.

**Table 3:** LULC area changes in the Aral Sea Basin from 1995 to 2025.

	Lulc	Cropland	Forestland	Grassland	Wetland	Urban	Bare land	Water bodies	Total
Area (*10^4^ha)	1995	2968.61	182.30	5644.28	7.56	12.07	7676.81	640.56	17132.19
	2000	2996.22	180.31	5650.10	7.59	13.62	7714.14	570.21	17132.19
	2005	2991.08	179.57	5690.24	7.59	33.36	7758.07	472.29	17132.19
	2010	2992.81	172.34	5702.90	7.58	44.90	7804.04	407.63	17132.19
	2015	2989.51	169.94	5706.75	7.56	59.52	7817.68	381.25	17132.19
	2020	2982.45	182.49	5722.59	7.54	72.69	7758.73	405.70	17132.19
	2025	2980.85	182.21	5741.17	7.52	78.07	7757.36	385.00	17132.19
Changes %	1995–2005	0.76	−1.50	0.81	0.33	176.31	1.06	−26.27	——
	2005–2015	−0.05	−5.36	0.29	−0.44	78.39	0.77	−19.28	——
	2015–2025	−0.29	7.22	0.60	−0.42	31.17	−0.77	0.98	——
	1995–2025	0.41	−0.05	1.69	−0.54	84.53	1.04	−66.38	——

**Table 4:** ESVs of the Aral Sea Basin from 1995 to 2025.

LULC	ESV (billion USD)				Changes (%)			
	1995	2005	2015	2025	1995–2005	2005–2015	2015–2025	1995–2025
**Cropland**	165.10	166.73	166.68	168.24	0.99	−0.03	0.94	1.90
**Forestland**	5.77	5.67	5.36	5.02	−1.66	−5.52	−6.23	−12.87
**Grassland**	235.37	235.58	237.72	243.48	0.09	0.91	2.42	3.45
**Wetland**	1.93	1.95	1.95	1.94	0.98	−0.13	−0.46	0.39
**Urban**	0.73	1.47	3.27	5.91	100.77	121.87	80.99	706.21
**Bare land**	0.00	0.00	0.00	0.00	0.00	0.00	0.00	0.00
**Water bodies**	83.65	69.27	48.00	43.37	−17.19	−30.71	−9.64	−48.16
**Total**	492.55	480.67	462.97	467.97	−2.41	−3.68	1.08	−4.99

We further analyzed the ESV changes in the upper, middle, and lower reaches of the Aral Sea Basin (Table S5). From 1995 to 2025, the upstream ESVs are predicted to fluctuate the least, with an overall reduction of 1.9 billion USD. The ESVs in the midstream will continue to increase, with a total increase of 6.82 billion USD and, except for an observed slight decrease in the wetland ESVs, the ESVs of the other land use types will increase. Among the increased ESVs, the grassland ESV increase, reaching 43.47%, accounted for the largest proportion, followed by urban land and cultivated land at 34.41% and 15.03%, respectively (Table S5). The downstream ESVs showed a continuous decrease, with a total reduction of 27.79 billion USD. Despite the reduction in ESVs associated with water bodies (−29.79 billion USD) and forestland (−0.04 billion USD) areas in the downstream regions, the ESVs of other land use types increased to varying degrees.

### Changes in ecosystem service function values

**Figure 7:**
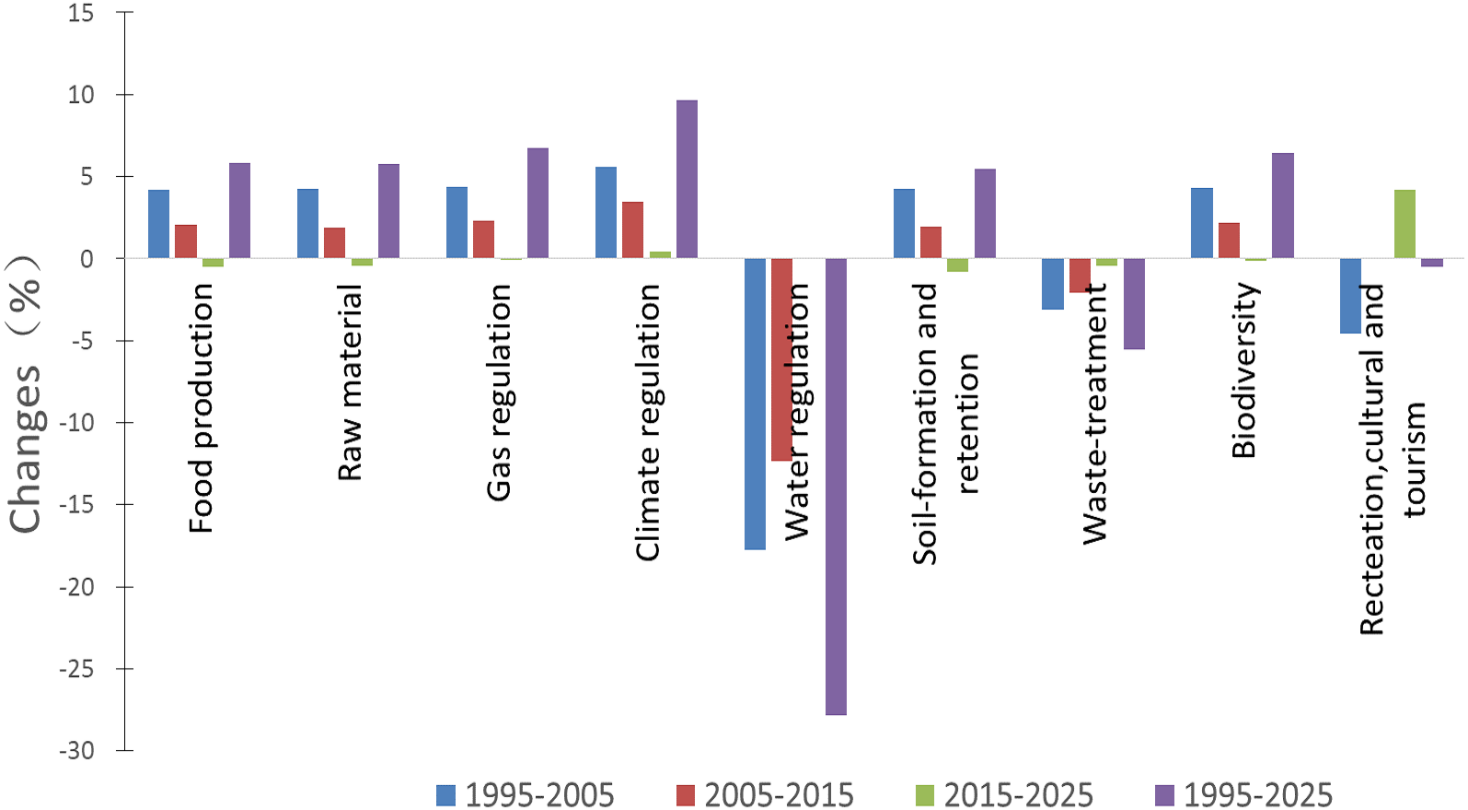
Change rate of ecosystem service functions in the Aral Sea Basin from 1995 to 2025.

Table 5 shows the individual ecosystem function values (ESV_ki_) of different land use types. The most important ecosystem functions in the Aral Sea Basin were biodiversity, food production, and water regulation, which contributed 35.9%, 28.12%, and 15.53%, respectively, of the total ESVs in 1995; 37.45%, 29.29%, and 12.78% in 2005; and 38.27%, 29.89%, and 11.2% in 2015. They are expected to contribute 38.21%, 29.75%, and 11.21%, respectively, of the total ESVs in 2025. The relative contributions of biodiversity and food production to regional ESVs have been increasing and overcompensated for losses in water regulation. Most of the ESV_ki_ were projected to increase between 1995 and 2025, except for water regulation, waste treatment, and culture and tourism, which decreased by 31.21%, 9.96%, and 5.17%, respectively. Across all ecosystem functions, water regulation had the highest change rate (Fig. 7).

**Table 5:** Estimated values for different ecosystem functions in the Aral Sea Basin from 1995 to 2025.

Service type	Sub-type	Ecosystem service value (billion USD )			
		1995	2005	2015	2025
Provisioning	Food production	137.51	138.39	138.43	138.68
	Raw material	9.92	9.99	9.98	10.00
Regulating	Gas regulation	0.51	0.51	0.51	0.52
	Climate regulation	15.11	15.41	15.63	15.79
	Water regulation	75.95	60.38	51.88	52.24
Supporting	Soil-formation and retention	22.09	22.25	22.24	22.21
	Waste-treatment	22.35	20.92	20.08	20.12
	Biodiversity	175.57	176.93	177.22	178.12
Culture	Recreation, cultural and tourism	30.02	27.68	27.14	28.47
Total		489.01	472.47	463.11	466.14

### Ecosystem sensitivity analysis

Although the wetland and bare land areas had the highest service value coefficients, the CS of the wetland and bare land areas was 0 because the wetland area was the smallest and the value of the bare land was zero. Meanwhile, the CS for the cropland increased from 0.34 to 0.36, and that of the grassland increased from 0.48 in 1995 to 0.51 in 2025 (Table 6). Compared with the grassland and cropland, the CS for the water body areas decreased from 0.16 to 0.1. In this study, all CS values were far smaller than “1”, indicating that the total estimated ecosystem value was inelastic with respect to the ecosystem value coefficients.

**Table 6:** Percentage change in the estimated total ESVs and coefficient of sensitivity.

Change of value coefficient	1995		2005		2015		2025	
	%	CS	%	CS	%	CS	%	CS
Cropland VC ± 50%	16.90	0.34	17.62	0.35	17.97	0.36	17.80	0.36
Forestland VC ± 50%	0.58	0.01	0.60	0.01	0.58	0.01	0.61	0.01
Grassland VC ± 50%	24.04	0.48	25.09	0.50	25.67	0.51	25.66	0.51
Wetland VC ±5 0%	0.20	0.00	0.21	0.00	0.21	0.00	0.21	0.00
Urban VC ± 50%	0.08	0.00	0.24	0.00	0.43	0.01	0.56	0.01
Bare land VC ± 50%	0.00	0.00	0.00	0.00	0.00	0.00	0.00	0.00
Water bodies VC ± 50%	8.19	0.16	6.25	0.13	5.15	0.10	5.17	0.10

### Analysis of the synergy degree of ecosystems trade-offs

After calculating the value of each ecosystem service in the Aral Sea Basin using the correlation analysis model, we obtained the correlations of nine types of ecosystem services (Table S6). When the result was positive, it indicated the presence of a synergistic relationship between two ecosystem services, *i.e.,* two targeted ecosystem services had the same upward or downward trend in the same period of time, and an increase in one service promoted an increase in the other. When the result was negative, there was a trade-off relationship between the two ecosystem services, *i.e.,* an increase in one ecosystem service led to a decrease in the other.

**Figure 8:**
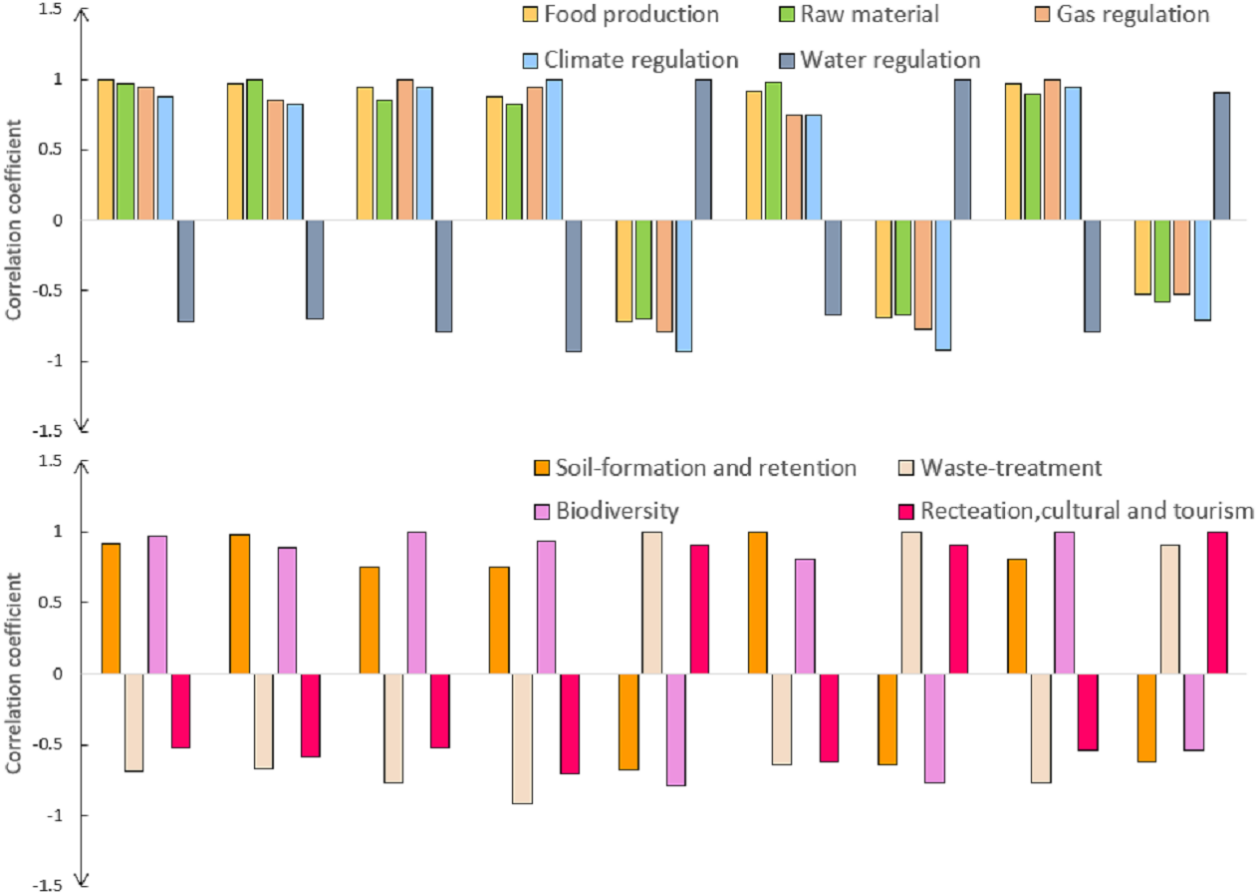
Interaction of ecosystem services in the Aral Sea Basin from 1995 to 2025.

Among the 81 values representing the nine ecosystem services in the Aral Sea Basin, 36 were smaller than 0 and 45 were greater than 0 (Table S6), which suggested that a synergistic relationship was dominant (55.56%). All of the trade-off relationships in the Aral Sea Basin were related to water regulation, waste treatment and prescription, and culture and tourism, but there was a strong synergy among all of them (Fig. 8). Additionally, strong synergy (*R* > 0.9; *p* < 0.01) was identified pairwise between food production-raw material, climate regulation-gas regulation, soil formation retention-raw material, waste treatment-water regulation, biodiversity-food production, biodiversity-gas regulation, and biodiversity-climate regulation (Table S6). Significant negative correlations (R < −0.9; *p* < 0.01) were also found between pairwise water regulation-gas, water regulation-climate, waste treatment-gas, waste treatment-climate, biodiversity-water, and biodiversity-water regulation.

## Discussion

### Impact of LULC change on ecosystem services in the Aral Sea Basin

Land use changes are caused and accelerated by many factors, including rapid urban expansion and cultivated land reclamation initiated by population expansion (Nahuelhual et al., 2014). In arid regions, water is the main factor affecting surface land use changes, and the use and distribution of water resources also directly lead to changes in ecosystem services (Kreuter et al., 2001). By analyzing the land use changes and calculating the ESVs of the Aral Sea Basin, we found significant urban area expansion (+599.22%) and the largest area increase from 1995–2025 can be attributed to grassland areas, which increased the ESVs of the Aral Sea Basin by 4.4 billion USD and 4.04 billion USD, respectively. Correspondingly, the area covered by water bodies was predicted to shrink the most (−39.9%), by 255.55 × 10^4^ ha from 1995 to 2025, causing a loss of 31.97 billion USD from the ESVs. The forestland area also shrank (0.09 × 10^4^ ha), resulting in an ESV loss of 2.73 million USD. Additionally, the cropland ecosystem service value increased by 0.68 billion USD from 1995 to 2025, which was mainly caused by the expansion of cropland areas (Table 4). Changes in the land use types of other areas were small.

To determine the partitioning and usage of water resources in the Aral Sea Basin, we calculated the changes in land use and ESVs in the upper, middle, and lower reaches of the Aral Sea Basin. The results showed that in the upper reaches of the Aral Sea Basin, where the LULC changed significantly, included urban land (+622.39%), waterbodies (−10.93%), and cropland (−1.83%) (Table S4). However, due to the small proportion in the total land area of the upper reaches, the ESVs of the upper reaches only slightly decreased (−1.9 billion USD) (Table S5). From 1995 to 2025, the ESVs of the middle reaches of the Aral Sea Basin will continue to increase. Except for a small decrease in the wetland ESVs, the ESVs of the other land use types increased to varying degrees. Urban, grassland, and cropland areas increased the most, by 3.78 billion USD, 1.79 billion USD, and 1.07 billion USD, respectively (Table S5). In this region, the bare land area decreased by 121.58 × 10^4^ ha, and the wetlands decreased by only 0.03 × 10^4^ ha. The fastest growth rates were found in urban (+536.99%) and forestlands (+24.57%) (Table S4). Unlike the upstream and midstream ESVs, the downstream ESVs experienced a drastic reduction. The ESVs of the water body areas experienced the largest reduction, accounting for 61.37% of the total ESVs reduction in the region, with a total reduction of 29.79 billion USD. However, the ESVs of the other land use types increased to varying degrees, including cropland (+0.46 billion USD), grassland (+1.18 billion USD), and urban land (+0.39 billion USD) (Table S5).

In comparison, the cropland, grassland, and urban land areas in the Aral Sea basin continued to increase, especially in the middle reaches, and accounted for 70.18%, 44.34%, and 86.02% of the total increase, respectively. With the continuous expansion of urban land, cropland, and grassland areas, water consumption continued to increase. The forestland and water body areas in the upstream and downstream reaches decreased, but they increased in the middle reaches. We believe that the midstream area intercepted a large amount of water from the Syr Darya and Amu Darya that allowed it to reclaim the bare land and develop cropland, grassland, and forestland areas through higher water consumption and building reservoirs, which has intensified the shrinkage of the Aral Sea Basin.

More cropland areas can produce more food to feed more people and bring considerable economic benefits. However, the Aral Sea Basin has an arid-semiarid climate, and the development of cropland consumes many water resources. Although more cropland brings considerable economic benefits, it can also result in the loss of natural ecosystem services (Li et al., 2019; Shi et al., 2020). In the Aral Sea Basin area, the original water body became bare land, which directly led to a rapid reduction in ESVs in this area. In the middle reaches of the region, the ESVs increased due to the expansion of cropland, grassland, and urban areas, but it could not compensate for the loss of downstream ESVs. At the same time, we projected that the expansion of agricultural land largely will lead to an increase in service functions related to food production (+1.2 billion USD), biodiversity (+2.5 billion USD), climate regulation (+0.7 billion USD), and soil formation (+0.1 billion USD) between 1995 and 2025 in the Aral Sea Basin (Table 5). However, the results show that agriculture and urban expansion have a negative impact on the management of other important ecosystem services, such as water regulation (−23.7 billion USD), waste treatment (−2.2 billion USD) and recreation, and culture and tourism (−1.6 billion USD).

Additionally, LULC changes are ecological processes dominated by temperature and precipitation, and climate change can also cause changes in land use types, which indirectly lead to changes in ESVs (Zhou et al., 2015). For example, the warming trends observed in Central Asia initially enhanced vegetative greenness before 1991, resulting in the enhancement of climate regulation in this region (Zhou et al., 2015). In recent years, however, precipitation in the Aral Sea basin has slightly decreased, the temperature has increased significantly, and the increased surface evaporation has put more pressure on the water resources in the region (Jiang et al., 2017), which will further affect LULC changes and the resulting ecosystem services.

### Analyzing the cooperative relationship of ecosystem services

The Aral Sea Basin is located in an inland arid area with diverse landform types, scarce rivers, and a unique relationship between various ecosystems. According to our results, there is a dominant synergistic relationship between the various ecosystem services in the Aral Sea Basin. A trade-off relationship exists between the ecosystem services of water regulation, waste treatment and recreation, culture and tourism, among others. However, these three ecosystem services (water regulation, waste treatment and recreation, culture and tourism) are synergistic in pairs.

In arid areas, water is a scarce resource that has unparalleled advantages in cultural tourism services. Additionally, water has a purification function and water in the Aral Sea Basin dominates almost all ecological processes. Since almost all of the nine ecosystem services (except for waste treatment and recreation, and culture and tourism) require water, they all have a trade-off relationship with water regulation. There are similar reasons behind the exceptions of waste treatment and recreation, and culture and tourism.

Because most of the land in the Aral Sea Basin is bare desert, increasing food production can increase the coverage of surface vegetation. Therefore, there is a synergistic relationship between raw material production, gas regulation, soil-formation retention, and biodiversity. The raw material designation generally refers to areas with a higher proportion of surface area covered by forests with strong soil retention capabilities. Surface vegetation cover has a strong effect on climate regulation, especially in arid areas. Areas with strong climate regulation functions also have high vegetation coverage and diverse plant species, so there is a strong synergistic relationship between climate regulation and biodiversity. In the same way, we also observed a strong synergy between climatic and atmospheric regulation and biodiversity.

### Protection policies for the Aral Sea basin

The reduction and irrational distribution of water resources in the Aral Sea Basin are the main factors decreasing the regional ESVs. Therefore, the protection and suitable use of water resources should be the focus of conservation efforts. Increases in population growth and social development will encourage urban land areas to continue to expand, and urban expansion will take up a large amount of arable land. To maintain agricultural production, more arable land can be developed from bare land and grassland areas, which consume more water resources. However, the Aral Sea Basin encompasses seven countries, and the coordinated use of water resources is particularly important. We suggest that the Aral Sea Conservation Organization distribute water resources from the Syr Darya and Amu Darya basins in a fair and reasonable way in order to prevent overexploitation.

Additionally, environmental protection policies for each country in this area should mainly focus on coordinating the relationship between cultivated and urban land areas, encouraging the return of unfavorable cultivated land to grassland, and preventing the further conversion of grassland or bare land to cultivated land in order to prevent the consumption of more water. First, permanently cultivated land should be designated as such in order to avoid the encroachment of built-up areas. This designation should not change under any circumstances, especially in areas with fertile soils. Otherwise, more arable land would need to be cultivated to produce the same yield, which would require more water consumption. Second, greater fees should be imposed for converting cultivated land into built-up areas. These fees should be used to subsidize farmers so that they may install more drip irrigation systems. Finally, to encourage the integration of rural farmland, certain types of crops should be planted according to their distance from a water source. Plants that consume a large amount of water, such as cotton and rice, should be cultivated on arable land that is close to rivers in order to prevent water loss during transportation.

### Limitations and areas of future research

In this study, the calculation of ESVs depended on changes in land use types, and the accuracy of determining land use types using remote sensing methods was affected by many factors, such as classification methods and data resolution (Li et al., 2019). These factors may be inaccurate during land use classification, which may lead to the underestimation or overestimation of ESVs (Sexton et al., 2015). During the evaluation process, we divided 37 land use types into seven categories (Table S1), among which shrubs were classified as grasslands, which may have caused an overestimation of ESVs.

When simulating land use, we chose the newly-proposed PLUS model, which takes into account many factors affecting land use distribution and uses Random Forest learning algorithms to analyze the impact of different influencing factors on land use. The reliability of the model was verified. As indicated by the results of the model validation and comparison, the simulation results obtained higher simulation accuracy (Kappa: 0.92; FoM: 0.24) than other land use simulation models, which was similar to the results of Liang et al. (2021). However, the PLUS model cannot simulate the evolution of land use composition and mixed structure at the sub-cell scale. Additionally, the premise of our prediction was that the region’s land use policies will not change (Zhang et al., 2020). These factors and assumptions may have caused deviations between the forecast results and the actual future conditions (López-Marrero et al., 2011).

Indeed, when addressing land-use conversion, we only assessed the responses of ecosystem services to changes in the area constituting a given land-use type. There was no discussion of the micromechanisms behind any changes in ecosystem services as part of the land use conversion process (Song & Deng, 2017), and no analysis of the socioeconomic response to land use changes was performed. Water from the Aral Sea is used to reclaim arable land to irrigate crops (considered a reasonable use), which can produce more food to feed more people. Future studies should explore which approach provides more social and economic benefits when protecting the Aral Sea.

## Conclusion

LULC changes in the Aral Sea Basin are predicted to change substantially during the 1995–2025 period due to the combined effects of social and economic development. As a result, 22.87 billion USD of ESVs will be lost. Our research shows that most of the lost ESVs were caused by the conversion of water bodies to bare land areas (98.19%). Through the analysis of land use and ESVs in the upper, middle, and lower reaches, we found that the ESVs of the middle reaches of the region increased, mainly due to the consumption of a large amount of water resources used there to reclaim bare land, irrigate farmland, and build towns. This series of human activities can increase food production and enhance raw material supplies, but excessive cultivation of land will also lead to a reduction in other ecosystem services (*e.g.*, climate and water regulation).

At the same time, the added ESV in the middle reaches was far less than the ESV lost downstream. Since the Aral Sea basin encompasses seven countries, we propose that some protection measures for the basin as a whole be implemented. However, the protection of the Aral Sea basin requires joint efforts from every country in the region. In future studies, we will combine remote sensing data and national statistical data to evaluate the changes in the ecological service value and social benefits brought about by land use changes in the Aral Sea Basin. The region’s eco-environmental protection and socioeconomic development should be coordinated to achieve the sustainable development goals outlined by the United Nations.

## Supplemental Information

10.7717/peerj.12623/supp-1 Supplemental Information 1 Classification of vegetation functional types from European Space Agency Climate Change Initiative land cover maps

10.7717/peerj.12623/supp-2 Supplemental Information 2 Markov chain matrix of LULCs transition probabilities for the period 2005–2010

10.7717/peerj.12623/supp-3 Supplemental Information 3 LULC area in the Aral Sea Basin from 1995 to 2020

10.7717/peerj.12623/supp-4 Supplemental Information 4 Area of land use types in the upper, middle and lower reaches of the Aral Sea Basin

10.7717/peerj.12623/supp-5 Supplemental Information 5 Ecosystem service value in the upper, middle and lower reaches of the Aral Sea Basin from 1995 to 2025

10.7717/peerj.12623/supp-6 Supplemental Information 6 Interaction of ecosystem services in the Aral Sea Basins

## Additional Information and Declarations

### Data Availability

The following information was supplied regarding data availability: The raw measurements are available in the Supplementary Files.
