# Supplementary material for: Spatiotemporal changes, trade-offs, and synergistic relationships in ecosystem services provided by the Aral Sea Basin
Source: PeerJ. 2021 Dec 16;9:e12623. doi: 10.7717/peerj.12623 (PMC8684718; doi:10.7717/peerj.12623)
Supplement: Supplemental Information 1 [file peerj-09-12623-s001.docx]

**Table S1 Classification of vegetation functional types.**

| Code | Original LULC | LULC |
| --- | --- | --- |
| 10 | Cropland, rainfed | Cropland |
| 11 | Herbaceous cover |  |
| 12 | Tree or shrub cover |  |
| 20 | Cropland, irrigated or post-flooding |  |
| 30 | Mosaic cropland (>50%)/natural vegetation (tree, shrub, herbaceous cover) (<50%) |  |
| 40 | Mosaic natural vegetation (tree, shrub, herbaceous cover) (>50%)/cropland (<50%) |  |
| 50 | Tree cover, broadleaved, evergreen, closed to open (>15%) | Forestland |
| 60 | Tree cover, broadleaved, deciduous, closed to open (>15%) |  |
| 61 | Tree cover, broadleaved, deciduous, closed (>40%) |  |
| 62 | Tree cover, broadleaved, deciduous, open (15–40%) |  |
| 70 | Tree cover, needleleaved, evergreen, closed to open (>15%) |  |
| 71 | Tree cover, needleleaved, evergreen, closed (>40%) |  |
| 72 | Tree cover, needleleaved, evergreen, open (15–40%) |  |
| 80 | Tree cover, needleleaved, deciduous, closed to open (>15%) |  |
| 81 | Tree cover, needleleaved, deciduous, closed (>40%) |  |
| 82 | Tree cover, needleleaved, deciduous, open (15–40%) |  |
| 90 | Tree cover, mixed leaf type (broadleaved and needleleaved) |  |
| 100 | Mosaic tree and shrub (>50%)/herbaceous cover (<50%) |  |
| 110 | Mosaic herbaceous cover (>50%)/tree and shrub (<50%) |  |
| 120 | Shrubland | Grassland |
| 121 | Evergreen shrubland |  |
| 122 | Deciduous shrubland |  |
| 130 | Grassland |  |
| 140 | Lichens and mosses |  |
| 150 | Sparse vegetation (tree, shrub, herbaceous cover) (<15%) |  |
| 151 | Sparse tree (<15%) |  |
| 152 | Sparse shrub (<15%) |  |
| 153 | Sparse herbaceous cover (<15%) |  |
| 160 | Tree cover, flooded, fresh or brakish water | Wetland |
| 170 | Tree cover, flooded, saline water |  |
| 180 | Shrub or herbaceous cover, flooded, fresh/saline/brakish water |  |
| 190 | Urban areas | Urban |
| 200 | Bare areas | Bare land |
| 201 | Consolidated bare areas |  |
| 202 | Unconsolidated bare areas |  |
| 210 | Water bodies | Waterbodies |
| 220 | Permanent snow and ice |  |
