# Supplementary material for: Spatiotemporal changes, trade-offs, and synergistic relationships in ecosystem services provided by the Aral Sea Basin
Source: PeerJ. 2021 Dec 16;9:e12623. doi: 10.7717/peerj.12623 (PMC8684718; doi:10.7717/peerj.12623)
Supplement: Supplemental Information 2 [file peerj-09-12623-s002.docx]

**Table S2 Markov chain matrix of LULCs transition probabilities for the period 2005–2010.**

| LULC | Cropland | Forestland | Grassland | Wetland | Urban | Bare land | Waterbodies |
| --- | --- | --- | --- | --- | --- | --- | --- |
| Cropland | 0.87 | 0.01 | 0.08 | 0.00 | 0.01 | 0.03 | 0.00 |
| Forestland | 0.08 | 0.75 | 0.16 | 0.00 | 0.00 | 0.00 | 0.00 |
| Grassland | 0.05 | 0.01 | 0.88 | 0.00 | 0.00 | 0.06 | 0.00 |
| Wetland | 0.02 | 0.00 | 0.04 | 0.86 | 0.00 | 0.06 | 0.02 |
| Urban | 0.21 | 0.00 | 0.01 | 0.01 | 0.77 | 0.01 | 0.00 |
| Bare land | 0.01 | 0.00 | 0.05 | 0.00 | 0.00 | 0.93 | 0.01 |
| Waterbodies | 0.02 | 0.00 | 0.04 | 0.00 | 0.00 | 0.06 | 0.88 |
