# Supplementary material for: Spatiotemporal changes, trade-offs, and synergistic relationships in ecosystem services provided by the Aral Sea Basin
Source: PeerJ. 2021 Dec 16;9:e12623. doi: 10.7717/peerj.12623 (PMC8684718; doi:10.7717/peerj.12623)
Supplement: Supplemental Information 3 [file peerj-09-12623-s003.docx]

**Table S3. LULC area in the Aral Sea Basin from 1995 to 2020.**

|  | **Lulc** | **Cropland** | **Forestland** | **Grassland** | **Wetland** | **Urban** | **Bare land** | **Water bodies** | **Total** |
| --- | --- | --- | --- | --- | --- | --- | --- | --- | --- |
| Area  （*10^4^ha） | 1995 | 2968.61 | 182.30 | 5644.28 | 7.56 | 12.07 | 7676.81 | 640.56 | 17132.19 |
|  | 1996 | 2970.86 | 181.15 | 5642.58 | 7.57 | 12.44 | 7719.85 | 597.74 | 17132.19 |
|  | 1997 | 2973.86 | 180.61 | 5643.36 | 7.57 | 12.77 | 7723.92 | 590.10 | 17132.19 |
|  | 1998 | 2981.10 | 180.27 | 5642.33 | 7.57 | 13.07 | 7724.52 | 583.34 | 17132.19 |
|  | 1999 | 2995.21 | 181.46 | 5640.77 | 7.59 | 13.33 | 7720.92 | 572.92 | 17132.19 |
|  | 2000 | 2996.22 | 180.31 | 5650.10 | 7.59 | 13.62 | 7714.14 | 570.21 | 17132.19 |
|  | 2001 | 2987.57 | 179.96 | 5656.12 | 7.59 | 17.68 | 7720.89 | 562.37 | 17132.19 |
|  | 2002 | 2994.92 | 180.79 | 5654.83 | 7.59 | 22.11 | 7718.33 | 553.63 | 17132.19 |
|  | 2003 | 2993.11 | 180.81 | 5663.81 | 7.59 | 26.18 | 7780.79 | 479.90 | 17132.19 |
|  | 2004 | 2995.82 | 180.43 | 5685.08 | 7.59 | 30.29 | 7756.65 | 476.34 | 17132.19 |
|  | 2005 | 2991.08 | 179.57 | 5690.24 | 7.59 | 33.36 | 7758.07 | 472.29 | 17132.19 |
|  | 2006 | 2989.66 | 178.34 | 5692.78 | 7.59 | 35.92 | 7761.38 | 466.51 | 17132.19 |
|  | 2007 | 2992.16 | 175.85 | 5694.69 | 7.59 | 38.86 | 7781.38 | 441.66 | 17132.19 |
|  | 2008 | 2991.36 | 173.86 | 5697.92 | 7.59 | 41.12 | 7785.33 | 435.01 | 17132.19 |
|  | 2009 | 2991.57 | 173.54 | 5700.99 | 7.59 | 43.13 | 7834.45 | 380.92 | 17132.19 |
|  | 2010 | 2992.81 | 172.34 | 5702.90 | 7.58 | 44.90 | 7804.04 | 407.63 | 17132.19 |
|  | 2011 | 2992.35 | 171.95 | 5703.35 | 7.58 | 46.74 | 7814.57 | 395.65 | 17132.19 |
|  | 2012 | 2994.00 | 170.81 | 5706.23 | 7.58 | 49.06 | 7820.93 | 383.59 | 17132.19 |
|  | 2013 | 2990.59 | 170.43 | 5707.80 | 7.57 | 52.72 | 7819.98 | 383.10 | 17132.19 |
|  | 2014 | 2991.47 | 169.94 | 5706.85 | 7.56 | 57.29 | 7816.38 | 382.71 | 17132.19 |
|  | 2015 | 2989.51 | 169.94 | 5706.75 | 7.56 | 59.52 | 7817.68 | 381.25 | 17132.19 |
|  | 2016 | 2981.86 | 173.57 | 5704.59 | 7.54 | 59.52 | 7814.41 | 390.70 | 17132.19 |
|  | 2017 | 2977.63 | 174.99 | 5701.87 | 7.54 | 64.97 | 7814.57 | 390.62 | 17132.19 |
|  | 2018 | 2977.48 | 177.94 | 5711.02 | 7.54 | 67.29 | 7805.22 | 385.71 | 17132.19 |
|  | 2019 | 2980.69 | 181.98 | 5716.91 | 7.54 | 72.68 | 7745.30 | 427.09 | 17132.19 |
|  | 2020 | 2982.45 | 182.49 | 5722.59 | 7.54 | 72.69 | 7758.73 | 405.70 | 17132.19 |
