# Supplementary material for: Spatiotemporal changes, trade-offs, and synergistic relationships in ecosystem services provided by the Aral Sea Basin
Source: PeerJ. 2021 Dec 16;9:e12623. doi: 10.7717/peerj.12623 (PMC8684718; doi:10.7717/peerj.12623)
Supplement: Supplemental Information 4 [file peerj-09-12623-s004.docx]

**Table S4 Area of land use types in the upper reaches of the Aral Sea Basin.**

|  | **Lucc** | **Cropland** | **Forestland** | **Grassland** | **Wetland** | **Urban** | **Bare land** | **Water bodies** | **Total** |
| --- | --- | --- | --- | --- | --- | --- | --- | --- | --- |
|  | 1995 | 822.13 | 172.02 | 2597.43 | 0.70 | 0.53 | 543.29 | 169.43 | 4305.53 |
| Area | 2005 | 812.55 | 170.21 | 2616.42 | 0.70 | 1.02 | 535.10 | 169.50 | 4305.52 |
| （*104ha） | 2015 | 823.65 | 160.75 | 2614.49 | 0.70 | 2.05 | 534.42 | 169.46 | 4305.53 |
|  | 2025 | 807.12 | 171.25 | 2622.97 | 0.70 | 3.81 | 548.76 | 150.92 | 4305.53 |
|  | 1995-2005 | -1.16 | -1.05 | 0.73 | -0.01 | 94.27 | -1.51 | 0.04 | —— |
| Changes | 2005-2015 | 1.37 | -5.56 | -0.07 | 0.00 | 99.88 | -0.13 | -0.02 | —— |
| % | 2015-2025 | -2.01 | 6.53 | 0.32 | -1.23 | 86.04 | 2.68 | -10.94 | —— |
|  | 1995-2025 | -1.82 | -0.45 | 0.98 | -1.24 | 622.39 | 1.01 | -10.93 | —— |

**Table S4 Area of land use types in the middle reaches of the Aral Sea Basin.**

|  | **Lucc** | **Cropland** | **Forestland** | **Grassland** | **Wetland** | **Urban** | **Bare land** | **Water bodies** | **Total** |
| --- | --- | --- | --- | --- | --- | --- | --- | --- | --- |
|  | 1995 | 1784.65 | 6.89 | 2466.65 | 6.66 | 10.57 | 3912.95 | 83.13 | 8271.50 |
| Area | 2005 | 1814.98 | 7.71 | 2485.95 | 6.68 | 29.95 | 3840.83 | 85.42 | 8271.51 |
| （*104ha） | 2015 | 1797.88 | 7.61 | 2501.06 | 6.64 | 53.03 | 3818.96 | 86.32 | 8271.51 |
|  | 2025 | 1803.77 | 8.58 | 2509.61 | 6.63 | 67.34 | 3791.37 | 84.20 | 8271.50 |
|  | 1995-2005 | 1.70 | 11.94 | 0.78 | 0.26 | 183.36 | -1.84 | 2.75 | —— |
| Changes | 2005-2015 | -0.94 | -1.30 | 0.61 | -0.50 | 77.04 | -0.57 | 1.06 | —— |
| % | 2015-2025 | 0.33 | 12.75 | 0.34 | -0.22 | 26.97 | -0.72 | -2.46 | —— |
|  | 1995-2025 | 1.07 | 24.57 | 1.74 | -0.47 | 536.99 | -3.11 | 1.29 | —— |

**Table S4 Area of land use types in the lower reaches of the Aral Sea Basin.**

|  | **Lucc** | **Cropland** | **Forestland** | **Grassland** | **Wetland** | **Urban** | **Bare land** | **Water bodies** | **Total** |
| --- | --- | --- | --- | --- | --- | --- | --- | --- | --- |
|  | 1995 | 361.83 | 3.39 | 580.20 | 0.20 | 0.98 | 3220.57 | 387.99 | 4555.17 |
| Area | 2005 | 363.55 | 1.64 | 587.87 | 0.21 | 2.38 | 3382.14 | 217.37 | 4555.17 |
| （*104ha） | 2015 | 367.98 | 1.58 | 591.20 | 0.21 | 4.44 | 3464.31 | 125.46 | 4555.17 |
|  | 2025 | 369.96 | 2.38 | 608.58 | 0.20 | 6.92 | 3417.24 | 149.88 | 4555.16 |
|  | 1995-2005 | 0.47 | -51.58 | 1.32 | 4.04 | 144.25 | 5.02 | -43.98 | —— |
| Changes | 2005-2015 | 1.22 | -3.99 | 0.57 | 0.00 | 86.06 | 2.43 | -42.28 | —— |
| % | 2015-2025 | 0.54 | 51.04 | 2.94 | -3.90 | 56.01 | -1.36 | 19.46 | —— |
|  | 1995-2025 | 2.25 | -29.79 | 4.89 | -0.02 | 608.98 | 6.11 | -61.37 | —— |
