# Supplementary material for: Spatiotemporal changes, trade-offs, and synergistic relationships in ecosystem services provided by the Aral Sea Basin
Source: PeerJ. 2021 Dec 16;9:e12623. doi: 10.7717/peerj.12623 (PMC8684718; doi:10.7717/peerj.12623)
Supplement: Supplemental Information 5 [file peerj-09-12623-s005.docx]

**Table S5 Ecosystem service value in the upper reaches of the Aral Sea Basin from 1995 to 2025.**

| LULC | **ESV (billion US$)** | | | | | **Changes (%)** | | | |
| --- | --- | --- | --- | --- | --- | --- | --- | --- | --- |
|  | 1995 | 2005 | 2015 | 2025 | 1995-2005 | | 2005-2015 | 2015-2025 | 1995-2025 |
| Cropland | 45.77 | 45.23 | 45.85 | 44.93 | -1.16 | | 1.37 | -2.01 | -1.82 |
| Forestland | 5.40 | 5.34 | 5.04 | 5.37 | -1.05 | | -5.56 | 6.53 | -0.45 |
| Grassland | 108.21 | 109.00 | 108.92 | 109.27 | 0.73 | | -0.07 | 0.32 | 0.98 |
| Wetland | 0.18 | 0.18 | 0.18 | 0.18 | -0.01 | | 0.00 | -1.23 | -1.24 |
| Urban | 0.04 | 0.07 | 0.14 | 0.25 | 94.27 | | 99.88 | 86.04 | 622.39 |
| Bare land | 0.00 | 0.00 | 0.00 | 0.00 | 0.00 | | 0.00 | 0.00 | 0.00 |
| Water bodies | 21.20 | 21.21 | 21.20 | 18.88 | 0.04 | | -0.02 | -10.94 | -10.93 |
| Total | 180.79 | 181.03 | 181.34 | 178.89 | 0.13 | | 0.17 | -1.35 | -1.05 |

**Table S5 Ecosystem service value in the middle reaches of the Aral Sea Basin from 1995 to 2025.**

| LULC | **ESV (billion US$)** | | | | | **Changes (%)** | | | |
| --- | --- | --- | --- | --- | --- | --- | --- | --- | --- |
|  | 1995 | 2005 | 2015 | 2025 | 1995-2005 | | 2005-2015 | 2015-2025 | 1995-2025 |
| Cropland | 99.35 | 101.04 | 100.09 | 100.42 | 1.70 | | -0.94 | 0.33 | 1.07 |
| Forestland | 0.22 | 0.24 | 0.24 | 0.27 | 11.94 | | -1.30 | 12.75 | 24.57 |
| Grassland | 102.76 | 103.56 | 104.19 | 104.55 | 0.78 | | 0.61 | 0.34 | 1.74 |
| Wetland | 1.71 | 1.71 | 1.71 | 1.70 | 0.26 | | -0.50 | -0.22 | -0.47 |
| Urban | 0.70 | 2.00 | 3.53 | 4.49 | 183.36 | | 77.04 | 26.97 | 536.99 |
| Bare land | 0.00 | 0.00 | 0.00 | 0.00 | 0.00 | | 0.00 | 0.00 | 0.00 |
| Water bodies | 10.40 | 10.69 | 10.80 | 10.54 | 2.75 | | 1.06 | -2.46 | 1.29 |
| Total | 215.14 | 219.24 | 220.56 | 221.96 | 1.91 | | 0.60 | 0.63 | 3.17 |

**Table S5 Ecosystem service value in the lower reaches of the Aral Sea Basin from 1995 to 2025.**

| LULC | **ESV (billion US$)** | | | | | **Changes (%)** | | | |
| --- | --- | --- | --- | --- | --- | --- | --- | --- | --- |
|  | 1995 | 2005 | 2015 | 2025 | 1995-2005 | | 2005-2015 | 2015-2025 | 1995-2025 |
| Cropland | 20.14 | 20.24 | 20.49 | 20.60 | 0.47 | | 1.22 | 0.54 | 2.25 |
| Forestland | 0.11 | 0.05 | 0.05 | 0.07 | -51.58 | | -3.99 | 51.04 | -29.79 |
| Grassland | 24.17 | 24.49 | 24.63 | 25.35 | 1.32 | | 0.57 | 2.94 | 4.89 |
| Wetland | 0.05 | 0.05 | 0.05 | 0.05 | 4.04 | | 0.00 | -3.90 | -0.02 |
| Urban | 0.07 | 0.16 | 0.30 | 0.46 | 144.25 | | 86.06 | 56.01 | 608.98 |
| Bare land | 0.00 | 0.00 | 0.00 | 0.00 | 0.00 | | 0.00 | 0.00 | 0.00 |
| Water bodies | 48.55 | 27.20 | 15.70 | 18.75 | -43.98 | | -42.28 | 19.46 | -61.37 |
| Total | 93.08 | 72.19 | 61.21 | 65.29 | -22.44 | | -15.21 | 6.66 | -29.86 |
