# Supplementary material for: Spatiotemporal changes, trade-offs, and synergistic relationships in ecosystem services provided by the Aral Sea Basin
Source: PeerJ. 2021 Dec 16;9:e12623. doi: 10.7717/peerj.12623 (PMC8684718; doi:10.7717/peerj.12623)
Supplement: Supplemental Information 6 [file peerj-09-12623-s006.docx]

**Table S6 Interaction of ecosystem services in the Aral Sea Basins**

| **Correlation** | **Food production** | **Raw material** | **Gas regulation** | **Climate regulation** | **Water regulation** | **Soil-formation retention** | **Waste-treatment** | **Biodiversity** | **Recreation, cultural and tourism** |
| --- | --- | --- | --- | --- | --- | --- | --- | --- | --- |
| Food production | 1.00 | .958^**^ | .865^**^ | .868^**^ | -.896^**^ | .846^**^ | -.884^**^ | .917^**^ | -.801^**^ |
| Raw material | .958^**^ | 1.00 | .707^**^ | .745^**^ | -.781^**^ | .933^**^ | -.762^**^ | .788^**^ | -.758^**^ |
| Gas regulation | .865^**^ | .707^**^ | 1.00 | .966^**^ | -.922^**^ | .473^**^ | -.924^**^ | .992^**^ | -.641^**^ |
| Climate regulation | .868^**^ | .745^**^ | .966^**^ | 1.00 | -.940^**^ | .530^**^ | -.941^**^ | .969^**^ | -.649^**^ |
| Water regulation | -.896^**^ | -.781^**^ | -.922^**^ | -.940^**^ | 1.00 | -.654^**^ | 1.000^**^ | -.935^**^ | .863^**^ |
| Soil-formation retention | .846^**^ | .933^**^ | .473^**^ | .530^**^ | -.654^**^ | 1.00 | -.632^**^ | ^**^.571^**^ | -.803^**^ |
| Waste-treatment | -.884^**^ | -.762^**^ | -.924^**^ | -.941^**^ | 1.000^**^ | -.632^**^ | 1.00 | -.934^**^ | .855^**^ |
| Biodiversity | .917^**^ | .788^**^ | .992^**^ | .969^**^ | -.935^**^ | .571^**^ | -.934^**^ | 1.00 | -.684^**^ |
| Recreation, cultural and tourism | -.801^**^ | -.758^**^ | -.641^**^ | -.649^**^ | .863^**^ | -.803^**^ | .855^**^ | -.684^**^ | 1.00 |

^**^P < 0.01; ^*^p <0.05
